# Microglia degrade Tau oligomers deposit via purinergic P2Y12-associated podosome and filopodia formation and induce chemotaxis

**DOI:** 10.1186/s13578-023-01028-0

**Published:** 2023-05-23

**Authors:** Subashchandrabose Chinnathambi, Rashmi Das

**Affiliations:** 1 Neurobiology Group, Division of Biochemical Sciences, CSIR-National Chemical Laboratory, Dr. Homi Bhabha Road, Pune, 411008 India; 2 Academy of Scientific and Innovative Research (AcSIR), Ghaziabad, 201002 India; 3 Department of Neurochemistry, National Institute of Mental Health and Neuro Sciences (NIMHANS), Institute of National Importance, Hosur Road, Bangalore, 560029 Karnataka India

**Keywords:** P2Y12, Migration, Filopodia, Podosome, Microglia, Tau oligomers

## Abstract

**Background:**

Tau protein forms neurofibrillary tangles and becomes deposited in the brain during Alzheimer’s disease (AD). Tau oligomers are the most reactive species, mediating neurotoxic and inflammatory activity. Microglia are the immune cells in the central nervous system, sense the extracellular Tau via various cell surface receptors. Purinergic P2Y12 receptor can directly interact with Tau oligomers and mediates microglial chemotaxis via actin remodeling. The disease-associated microglia are associated with impaired migration and express a reduced level of P2Y12, but elevate the level of reactive oxygen species and pro-inflammatory cytokines.

**Results:**

Here, we studied the formation and organization of various actin microstructures such as-podosome, filopodia and uropod in colocalization with actin nucleator protein Arp2 and scaffold protein TKS5 in Tau-induced microglia by fluorescence microscopy. Further, the relevance of P2Y12 signaling either by activation or blockage was studied in terms of actin structure formations and Tau deposits degradation by N9 microglia. Extracellular Tau oligomers facilitate the microglial migration via Arp2-associated podosome and filopodia formation through the involvement of P2Y12 signaling. Similarly, Tau oligomers induce the TKS5-associated podosome clustering in microglial lamella in a time-dependent manner. Moreover, the P2Y12 was evidenced to localize with F-actin-rich podosome and filopodia during Tau-deposit degradation. The blockage of P2Y12 signaling resulted in decreased microglial migration and Tau-deposit degradation.

**Conclusions:**

The P2Y12 signaling mediate the formation of migratory actin structures like- podosome and filopodia to exhibit chemotaxis and degrade Tau deposit. These beneficial roles of P2Y12 in microglial chemotaxis, actin network remodeling and Tau clearance can be intervened as a therapeutic target in AD.

**Supplementary Information:**

The online version contains supplementary material available at 10.1186/s13578-023-01028-0.

## Introduction

Alzheimer’s disease (AD) is a progressive neurodegenerative disease, which involves synaptic loss, neuroinflammation and neuronal death. AD is characterized by the extracellular deposition of amyloid-β (Aβ) plaques and intracellular neuro-fibrillary tangles (NFTs) of Tau protein [1, 2]. In the progressive stages of AD, the Tau protein becomes post-translationally modified which leads to oligomerization and further aggregation [3–5]. Disease-associated Tau species can propagate from damaged neurons to healthy neurons through various processes such as exosomes, membrane leakage, cell-to-cell junctions and neurotransmitter release etc*.* [6–8]. Microglia are the prime immune cells in the brain, which can sense the extracellular Tau via death-associated molecular pattern (DAMPs) receptors and become activated to clear misfolded proteins from parenchyma [9–12]. Microglia migrate at the site of neuronal damage and plaque deposits via sensing extracellular chemical gradient and mediate immune response [13, 14]. However, the senescent microglia or disease-associated microglia (DAMs) exacerbate the hyper-inflammation, reactive oxygen species (ROS) production, and complement-mediated faulty engulfment of synapses, which leads to neuronal death and cognitive loss [15–18].

Microglia alternate between two distinct phenotypes such as- ‘ramified’ (homeostatic) and ‘ameboid’ (activated), to mediate synaptic surveillance, neuronal health, neurotransmitter recycling, tissue homeostasis, migration, and pathogen recognition [19, 20]. Microglia remodel the membrane-associated actin network for chemotaxis, phagocytosis, endocytosis, and vesicular trafficking [21–23]. Actin remodeling consists of various microstructures such as lamellipodia, filopodia, podosome, invadopodia, focal adhesion-stress fiber, and cortical actin sheet [24, 25]. Lamellipodia produces firm anchorage onto the substratum and generates tensile mechanical force to move forward. However, filopodia involve in mechano-sensing, adhering, object trapping, and polarization [26]. Podosome are the short-lived, protrusive ventral actin structure, which is composed of a cross-linked actin core, surrounded by vinculin and adhesion receptor’s ring [27]. Podosome mediate various physiological functions, including matrix adhesion, degradation, migration, invasion, and many more that need to be explored further [28–30]. The Src kinase and TKS5 scaffold regulate the actin flux, nucleation, and podosome formation in physiological cell migration [31]. The previous report demonstrated the microglial migration in response to plaque accumulation, neuronal damage, and inflammation in neurodegenerative diseases [31]. Hence, the structural and functional organization of podosome and other actin protrusions need to be explored in the scenario of extracellular Tau-induced microglial migration.

Purinergic signaling plays a vital role in microglial chemotaxis, neuronal health maintenance, and tissue homeostasis [32]. Microglia form the P2Y12-mediated somatic junctions with neurons to surveil neuronal health [33]. Similarly, the induction of P2Y12 signaling leads to microglial process extension, but the P2Y12 depletion resulted in reduced brain surveillance, immune activation, IL1β secretion, and synaptic elimination [34–36]. P2Y12 signaling is related to Ca^2+^ signaling in migratory microglia where the podosome contains Ca^2+^ ion channels, Iba1 and calmodulin for directional movements [37, 38]. Previously, it was shown that the P2Y12 signaling and actin remodeling-associated microglial migration become hampered due to aging [39]. Therefore, the structure and function of this purinoceptor-P2Y12 in actin structures like- podosome, filopodia, and lamellipodia need to be explored in Tauopathy-associated migratory microglia.

Thus, our study focused on the localization of P2Y12 in podosome, filopodia, and uropod in Tau-induced migratory microglia. Moreover, the actin nucleation and podosome formation were depicted in terms of Arp2 and TKS5 colocalization within the F-actin-rich region. Thereafter, the effect of P2Y12 activation by ADP and blockage by Clopidogrel would elucidate the correlation of microglial migration, podosome and filopodia formation and also with Tau deposit degradation.

## Materials and methods

### Preparation of Tau monomer and oligomers and its characterization

Tau protein was expressed in *E. coli* BL21* and purified by cation exchange and followed by size-exclusion chromatography, as described previously [40]. Tau oligomers were prepared by inducing with polyanionic factor heparin (17.5 kDa) in PBS (pH 7.4) for 12 h, as described earlier [41]. Briefly, Tau oligomers were stabilized using 0.01% glutaraldehyde for 10 min. The oligomers were then buffer exchanged twice with PBS by centrifuging at 3200 rpm for 2 h with 10 kDa molecular cut-off filters. The concentrated oligomers were collected and the concentration was measured by BCA assay. Further, the quality of the oligomers and monomer were checked by 10% SDS-PAGE and stored at − 80 °C. The core-β sheet structures of Tau oligomers were characterized by Thioflavin-S (440/521 nm), and the exposed hydrophobic patches on Tau species were quantified by ANS fluorescence (375/490 nm) in fluorescence spectrophotometer (Infinite® 200 M PRO, Tecan). Tau oligomers were visualized by transmission electron microscopy (TEM) by spotting onto 400-mesh carbon-coated copper grids and stained with 2% uranyl acetate. The grids were dried and analyzed by Tecnai T20 at 120 kV for TEM. Tau oligomers and monomers were spotted onto the nitrocellulose membrane at equal concentrations and allowed for complete drying. The blots were blocked with 5% skimmed milk in PBS and probed with A11 antibody (1:1000 dilution) and K9JA antibody (1:8000 dilution) overnight and 1 h, respectively. The blots were probed with anti-rabbit secondary antibody, developed using ECL reagent in Amersham Imager 600.

### Tagging of Tau monomer and oligomers by Alexa^647^- C2 maleimide

The 100 µM of Tau monomer was diluted in PBS and incubated with 10 molar excess TCEP (tris(2-carboxyethyl)phosphine) for 10 min while Tau oligomers were diluted only in PBS. Then, Tau monomer and oligomers were mixed with Alexa^647^-C2 maleimide drop-wise at 2 molar excess concentrations and incubated overnight at 4 °C in 600 rpm shaking condition. After incubation, the unbound Alexa^647^ was removed by buffer-exchange twice with PBS in 3 KDa molecular cut-off filters by centrifuging at 12000 rpm for 5 min. The Alexa^647^ tagged Tau monomer, and oligomers were collected separately and the concentrations were checked by BCA assay and characterized by SDS-PAGE, Thioflavin-S, ANS fluorescence, and TEM study.

### Western blot

To study the expression level of Arp2, TKS5 upon Tau exposure, the N9 cells (3X10^6^ cells/treatment group) were treated with Tau monomer and oligomers at a concentration of 45 μg/ml (1 μM), with ADP at 50 μM concentration, as a positive control of P2Y12 activation, for 24 h. The cells were washed with PBS and lysed with RIPA buffer. The cell lysates were subjected to Bradford assay, and 75 μg of cell lysates were processed for western blot with anti-Arp2 and TKS5 antibody (1:1000 dilution) with anti-α/β-tubulin antibody (1:5000 dilution) as a loading control. The band intensity for target proteins and loading control protein among various groups was quantified by using BIORAD Quality one 4.6.6 software. The band density of the treated group was compared with cell control group and normalized with the loading control (α/β-tubulin) (n = 3). Then, the relative fold changes for the target proteins were plotted in comparison with housekeeping control.

### Immunofluorescence Study

The Arp2, TKS5 and P2Y12-associated actin remodeling on microglia upon Tau exposure (45 µg/ml) were checked by Immunofluorescence study. The N9 cells (25000 cells) were treated with Tau monomer, oligomers and ADP (50 µM) for 24 h. In the time-dependent TKS5-localized podosome accumulation experiment, N9 microglia were treated with Tau oligomers (45 µg/ml) along with cell control from 1 to 12 h time points. After incubation, the cells were washed with PBS and fixed with 4% paraformaldehyde for 15 min, permeabilized with 0.2% TritonX-100 and blocked with 2% horse serum in PBS buffer. The cells were stained with P2Y12 (1:100), Arp2 (1:100), TKS5 (1:100) antibody and phalloidin-alexa^488^ (1:40) for overnight at 4 °C. Then, alexa flour-tagged secondary antibodies were allowed to bind P2Y12, Arp2 and TKS5 for 1 h along with nuclear stain-DAPI (300 nM). The microscopic images were taken in Zeiss Axio observer with Apotome2 fluorescence microscope at 63X oil immersion objective. The quantification for mean fluorescence intensity (n = 50) and area of cells (n = 40) was done using ZEN 2.3 software and plotted for different test groups. The numbers of podosome^+^ (n = 13 fields) and filopodia^+^ (n = 22 fields) N9 cells, % of cells with different podosome rearrangements (n = 28), time kinetics podosome formation (n = 10 fields) were counted in and plotted.

For the determination of 2D microglial migration upon P2Y12 activation and blockage, the N9 cells (5X10^6^ cells) were treated with 45 µg/ml concentration of Tau monomer, oligomers, along with ADP (50 μM) and Clopidogrel (2 μM). At first, 3 scratches per group were made with 200 µl tips, washed, and then, Tau monomer and oligomers were added separately along with ADP and Clopidogrel and incubated for upto 24 h. The phase-contrast images were taken at various time intervals from 0 to 24 h in Zen Axio observer 7 microscope at 20X magnification. The wound lengths were measured in different positions at 24 h time intervals and the % of wound closure was calculated in comparison with cell control. The experiment was performed thrice, and multiple values were taken from single groups for wound closure (n = 12).

### Trans-well migration assay

N9 microglia were subjected to trans-well migration in response to extracellular Tau species along with ADP and Clopidogrel as a determinant of P2Y12-related chemotaxis. N9 cells (50,000 cells/inserts), were treated with ADP and Clopidogrel, and were seeded in the upper chamber of 24-well plate format. While, the Tau monomer, oligomers were added at a concentration of 45 µg/ml in lower chamber for 24 h. The lower surface of the inserts containing migrated microglia was fixed with 4% paraformaldehyde and stained with 0.2% crystal violet solution and dried. The upper surface of the inserts containing non-migrated cells was removed by using a cotton swab and the lower surfaces were imaged at 20X objective under a bright-field microscope. The experiment was performed twice and the numbers of migrated cells per field were counted (n = 12 fields) in different treatment groups.

###  Tau deposit degradation assay

For the Tau deposit degradation assay, the 18 mm coverslips were coated with 10 μg/cm^2^ Alexa^647^ tagged-Tau protein (monomer and oligomers) in PBS and incubated overnight at 4 °C. After incubation, the excess solution was aspirated by vacuum, and the coverslips were dried at 37 °C for 30 min. The coated coverslips were sterilized by UV exposure for 30 min in a laminar hood and washed with PBS twice. The Alexa^647^-tagged Tau (monomer and oligomers) coated coverslips were neutralized with 10% FBS containing RPMI media for 15 min. The N9 cells were seeded at a density of 50,000 cells/well for Tau degradation experiment. The cells were incubated for 8 h and 24 h for both Tau monomer and oligomers-deposits. After incubation, the cells were fixed with 4% paraformaldehyde and directly blocked with 2% horse serum, 0.2% TritonX-100 containing PBS for 1 h. The cells were stained with phalloidin-alexa^488^ (1:40), TKS5 (1:100), Arp2 (1:100), and P2Y12 (1:100) antibodies overnight at 4 °C. The anti-rabbit secondary antibody-Alexa^555^ was used to bind Arp2, TKS5, and P2Y12 for 1 h along with nuclear stain-DAPI (300 nM). The microscopic images were taken in Zeiss Axio observer with Apotome2 fluorescence microscope at 63X oil immersion objective. The quantification for mean fluorescence intensity (n = 100 cells) was done using ZEN 2.3 software and plotted for different test groups. The % cells with deposit degradation (n = 40 fields), the relative area of Tau degradation/total cell area (n = 45 fields) and P2Y12 activation/blockage-related Tau degradation (n = 30 fields) were counted in multiple fields. The colocalization analysis in podosome and filopodia-associated Tau deposit degradation (n = 25 cells) in treated groups in multiple fields.

### Statistical analysis

All experiments were performed in three biological replicates and each measurement for every experiment was taken in triplicate. Statistical analyses were performed for fluorometric assay and microscopic quantification by using one-way ANOVA. The statistical significance among various groups has been calculated by Tukey–Kramer’s analysis at 5% level of significance. For microscopic analysis, several data points from multiple fields were plotted. The test groups were compared with untreated cell control and the *p*-values were mentioned within the figures. In ADP or Clopidogrel-mediated actin remodeling and Tau deposit degradation experiment, the ADP/Clopidogrel + Tau species-treated groups were compared to ADP/Clopidogrel treated group as well as only monomer/oligomers groups (For *eg.* ADP + Tau monomer vs. ADP/ only monomer, Clopidogrel + oligomers vs. Clopidogrel/only oligomers etc*.*). Hence the *p*-values were quantified and depicted within the figures.

## Results

### Extracellular Tau oligomers induce Arp2-associated podosome and filopodia formation but reduce Arp2 from uropod.

Tau is a microtubule-associated protein that becomes oligomerized and further aggregated as neurofibrillary tangles in the progressive stages of AD [42]. The neuronal escape of Tau oligomers in the brain surroundings can activate the surveilling microglia to mediate migration, phagocytosis and immune response [43]. To understand the effect of Tau oligomers on microglial migration, we have prepared and characterized a stable globular Tau oligomer, which contained more β-sheet structures and surface hydrophobicity by ThS and ANS fluorescence, respectively, as compared to monomer (Additional file 1: Figure S1A–E). The Alexa^647^ tagged Tau oligomers were found to retain globular structures, as observed by TEM study and β-sheet structures by ThS fluorescence (Additional file 1: Figure S1F–I).

**Fig. 1:**
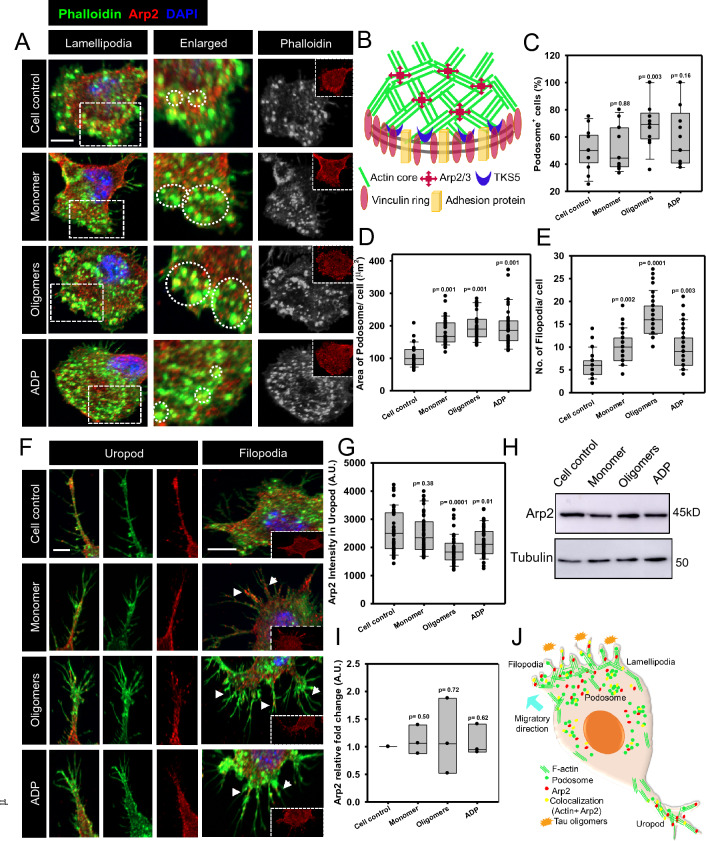
Tau oligomers induce podosome and filopodia formation, orchestrated with Arp2. **A** Extracellular Tau oligomers-induced podosome accumulation more in microglial lamellipodia, than Tau monomers as observed by immunofluorescence (IF) assay. The microglial podosome was found to be colocalized with actin-nucleator protein Arp2 for rapid actin polymerization in response to Tau oligomers similar to ADP exposure in migratory microglia (scale bar 10 μm). **B** A simplistic illustration of podosome where actin cross-linking is mediated by actin nuclear Arp2/3 complex. The actin cores are surrounded by the vinculin ring and adhesion protein receptors. The attachment of actin core fibers with membrane receptors is driven by adaptor protein TKS5. **C** The quantification of microscopic images showed that the exposure of Tau oligomers has significantly induced the podosome^+^ microglia by 70% as compared to the monomer-exposed group (50%). (No. of experiment = 3) (n = 13). **D** Moreover, podosome-associated area has increased almost twice in Tau-exposed microglial cells as compared to cell control. (No. of experiment = 3) (n = 40). **E** Tau oligomers have increased the number of filopodia three times more compared to untreated control microglia. While, the Tau monomer and ADP exposure have induced the filopodia numbers twice than the control population, as seen by microscopic image quantification. (No. of experiment = 3) (n = 50). **F** Similar to podosome, Tau oligomers facilitated the localization of Arp2 in filopodia and branched uropod in microglia, for actin polymerization during migration (scale bar 10 μm). **G** The Arp2 level was reduced from microglial uropod upon Tau oligomers exposure, relating rapid actin dynamics towards frontal lamellipodia. (No. of experiment = 3) (n = 50). **H**, **I**. The Arp2 expression level remained unaltered in various Tau and ADP exposure by Western blot analysis and relative fold change calculation in N9 microglia. (n = 3). **J** The extracellular Tau oligomers have the potential to induce the accumulation of podosome and filopodia by localizing Arp2 at lamellipodia of microglia, which plays an essential role in migration and mechanosensing

Previously, we showed that microglia remodel the membrane-associated actin network in response to extracellular toxic Tau species [41]. Here, we further showed that microglia displayed a specialized actin structure podosome, which was colocalized with actin-nucleator protein-Arp2, in response to extracellular Tau oligomers. The podosome was observed to accumulate more at the microglial lamellipodia upon Tau oligomers exposure than monomers (Fig. 1A). In schematic representation, the podosome contains a core of branched actin networks, surrounded by a vinculin ring. The nucleator Arp2/3 complex and TKS5 scaffold protein bind to the cross-linked actin network with adhesive membrane receptors (Fig. 1B). The extracellular Tau oligomers exposure induced the number of podosome-bearing microglia by 20% as compared to monomer and cell control (Fig. 1C). Also, the podosome-associated area was increased by Tau exposure in migratory microglia similar to ADP, as a positive regulator (Fig. 1D, Additional file 1: Figure S1J).

Extracellular Tau has induced the Arp2-decorated filopodia formation in microglia as compared to untreated control. Moreover, the number of filopodia was increased by two times in Tau monomer exposure and three times in oligomers exposure than cell control (Fig. 1E, F). The contractile rear end of the migratory cells is called the uropod [44]. The exposure of extracellular Tau oligomers was shown to form the branched uropod in microglia, emphasizing more cortical adhesions (Fig. 1F, Additional file 1: Figure S1K, L). The microscopic quantification depicted the reduced level of Arp2 in the microglial uropod upon the oligomer exposure, which may signify the rapid turnover of actin towards the lamella from the uropod (Fig. 1G). The western blot analysis showed that the Arp2 level remained unaltered in Tau-induced and ADP-induced microglial populations (Fig. 1H, I). Hence, the extracellular Tau oligomers facilitated the podosome and filopodia-associated actin polymerization by Arp2 via altering the actin turnover towards lamella from uropod during migration (Fig. 1J).

### Tau oligomers induce the accumulation of TKS5-localized podosome clusters at lamellipodia in a time-dependent manner

**Fig. 2:**
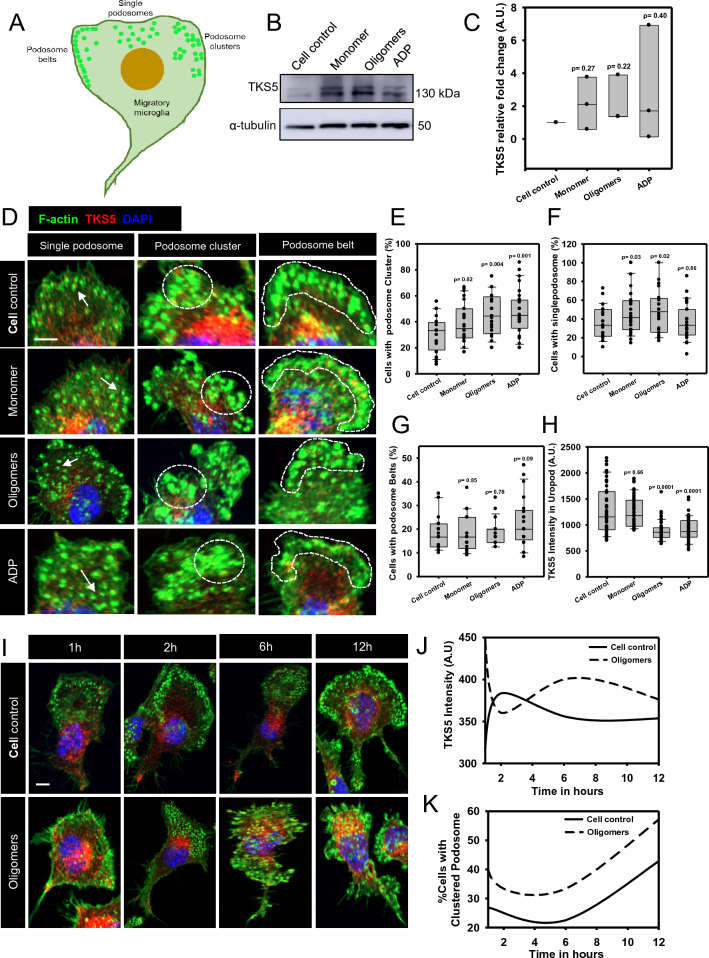
Extracellular Tau modulates the formation of TKS5-mediated podosome clusters in microglia. **A** Microglia rearrange the podosome differently at frontal lamellipodia such as podosome belts, clustered podosome, and single podosome, for the adherence to the substratum by membrane-associated actin polymerization. **B**, **C** Western blot analysis and relative fold change showed no significant changes in the expression of TKS5 protein upon various Tau exposure and ADP treatment in microglia (n = 3). **D** IF study revealed that microglia form various rearrangements of podosome upon exposure to extracellular Tau species and ADP. Among all structures, the podosome clusters were more evident in oligomer-treated microglia than in monomer exposure (scale bar 10 μm). **E** The clustered podosome accumulated by 20% more in lamellipodia upon Tau oligomers exposure, similar to ADP and cell control. (No. of experiment = 3) (n = 28). **F** While, The amount of single podosome arrangement in microglia remains unaltered among various Tau-treated groups. (No. of experiment = 3) (n = 28) **G** The podosome belts containing cells remain unaltered in Tau-induced migratory microglia. (No. of experiment = 3) (n = 28). **H** Furthermore, the TKS5 intensity of uropod was decreased in Tau oligomer-induced microglia, which may signify the rapid actin and actin-associated protein turnover from the rear end towards lamella upon migration. (No. of experiment = 3) (n = 50). **I**, **J** The time-dependent IF study showed that the extracellular Tau oligomers induced the accumulation of clustered podosome in microglial lamellipodia (scale bar 10 μm). Then, the quantification of microscopic images revealed that the TKS5 intensity was increased during the oligomers exposure from 6 h, compared to cell control. (No. of experiment = 3) (n = 40). **K** Further, the clustered podosome-containing microglial population have increased in a time-dependent manner in Tau oligomers-exposed microglia than to untreated cells. (No. of experiment = 3) (n = 10)

Various podosome rearrangements were witnessed in cells, such as podosome clusters, belts, rosettes, and single podosome (Fig. 2A). Nevertheless, the actual function of these particular rearrangements is not known [31]. TKS5 scaffold plays an important role in podosome formation and positioning the metalloproteases, while the loss of TKS5 leads toreduced podosome formation [45]. In our study, microglia showed the unaltered expression level of TKS5 in various Tau-treated groups (Fig. 2B, C), although the formation and area of the podosome have been increased significantly in Tau/ADP-exposed microglia. We observed that microglia formed various arrangements of podosome such as clusters, belts, and single upon extracellular stimuli like Tau oligomers or ADP (Fig. 2D, Additional file 1: Figure S2A). The quantification of microscopic images depicted that the podosome clusters-bearing microglial percentage significantly increased by 20% upon Tau oligomers and ADP exposure, as compared to cell control (Fig. 2E, Additional file 1: Figure S2B). While the other podosome arrangements, like-single and podosome belts, were elusive in Tau/ADP-induced microglia (Fig. 2F, G). Similar to Arp2, the TKS5 intensity was reduced in uropod, while the F-actin intensity remains unaltered in both podosome and filopodia in Tau oligomers and ADP-exposed microglia (Fig. 2H, Additional file 1: Figure S2C, D). These might emphasize the active podosome turnover towards lamellae from uropod during Tau-induced microglial migration (Additional file 1: Figure S2E). Similarly, the TKS5 intensity was found to be elevated after 4 h and the podosome cluster^+^ microglia population was increased from 6 h of Tau oligomers exposure (Fig. 2I-K, Additional file 1: Figure S3A). The 3D illustrations of microscopic images revealed the accumulation of podosome in microglial lamella at 12 h of Tau oligomers exposure as compared to untreated control (Additional file 1: Figure S3B). Hence, the microglia preferentially organize the TKS5-associated podosome clusters at lamellipodia for adhering to nascent sites and moving forward during Tau-induced migration.

### Tau oligomers orchestrate P2Y12 in podosome, filopodia and uropod

**Fig. 3:**
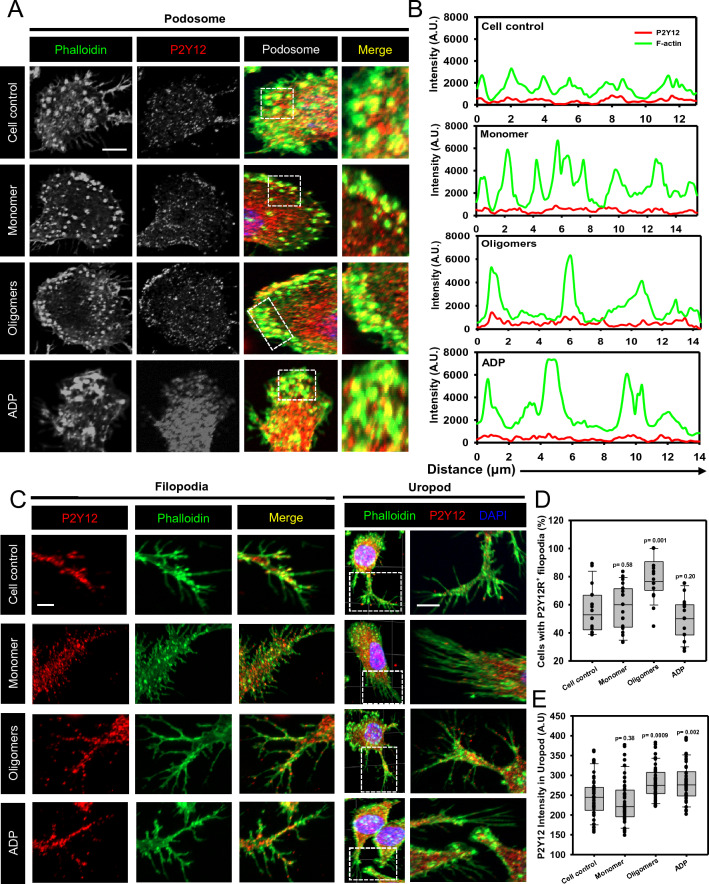
P2Y12-associated podosome modulates microglial migration and invasion. **A** Purinergic P2Y12 receptors were colocalized with F-actin-rich podosome in lamellipodia during Tau-induced microglia migration. Similarly, the activation of P2Y12 signaling by ADP has also induced the colocalization of P2Y12 in podosome-rich microglial lamella, as seen by the IF study (scale bar 10 μm). **B** Fluorescence quantification of podosome-rich lamella near the membrane (box marked area) showed the colocalization of P2Y12 and F-actin at the distances of 14 μm, in Tau monomer, oligomers-exposed microglia. **C**, **D** The wound scratch assay showed that the extracellular Tau and ADP, together and separately induced the microglial migration as quantified by %wound closure at 24 h. The blockage of P2Y12 signaling by Clopidogrel has reduced the microglial migration. But, Tau oligomers restored the microglial migration even upon clopidogrel-induced blockage, as observed and quantified by phase contrast imaging. (No. of experiment = 3) (n = 12) (Scale bar 100 μm). **E**, **F** In trans-well migration assay, Tau oligomers have induced the microglial invasion more than Tau monomer treatment. P2Y12 activation by ADP has induced microglial trans-migration in both Tau monomer and oligomers exposure. The quantification of microscopic images revealed that Clopidogrel exposure has significantly reduced the microglial invasion, which was eventually restored by Tau oligomers exposure. These emphasize that Tau oligomers were better chemoattractants and can intervene P2Y12 signaling during migration/invasion. (No. of experiment = 2) (n = 12) (scale bar 100 μm)

P2Y12 receptor is involved in ADP-mediated chemotaxis in microglia toward the site of neuronal damage and plaque deposits [46, 47]. Here, during the Tau oligomers-induced microglial migration, P2Y12 was found to be localized with actin core in the podosome at frontal lamellipodia (Fig. 3A). The quantification of microscopic images depicted the colocalization of F-actin and P2Y12 in membrane-associated podosome clusters in Tau-exposed microglia (Fig. 3B). Moreover, the extracellular Tau oligomers induced the P2Y12^+^ podosome as compared to the ADP-exposed microglial population (Additional file 1: Figure S3C). The 3D microscopic images revealed that Tau oligomers have induced the localization of P2Y12 in clustered podosome while monomer exposure induced the single podosome formation (Additional file 1: Figure S3D). This might suggest the existence of the P2Y12 purinoceptor which dictates the cellular directionality/ movements in podosome-mediated adhesion and migration.

Microglia display P2Y12-associated filopodia to migrate at the site of injury, while the P2Y12 mutation resulted in fewer filopodia formation and induced engulfment of damaged neurons in the epileptic brain [48, 49]. In our study, we found that extracellular Tau has induced the P2Y12-associated filopodia formation in migratory microglia. Similarly, the P2Y12 localization was observed to increase in branched uropod structures during Tau oligomers-induced migration (Fig. 3C). Microscopic quantification showed that extracellular Tau oligomers induced the number of cells containing P2Y12^+^ filopodia as compared to monomer-treated cells (Fig. 3D). Moreover, the localization of P2Y12 was increased in microglial rear ends- uropod (Fig. 3E), suggesting that the P2Y12 localizes in migration-associated actin structures- filopodia and uropod for increased cell adhesion and matrix sensing.

### The blockage of P2Y12 signaling reduces cell migration in Tau-induced microglia

**Fig. 4:**
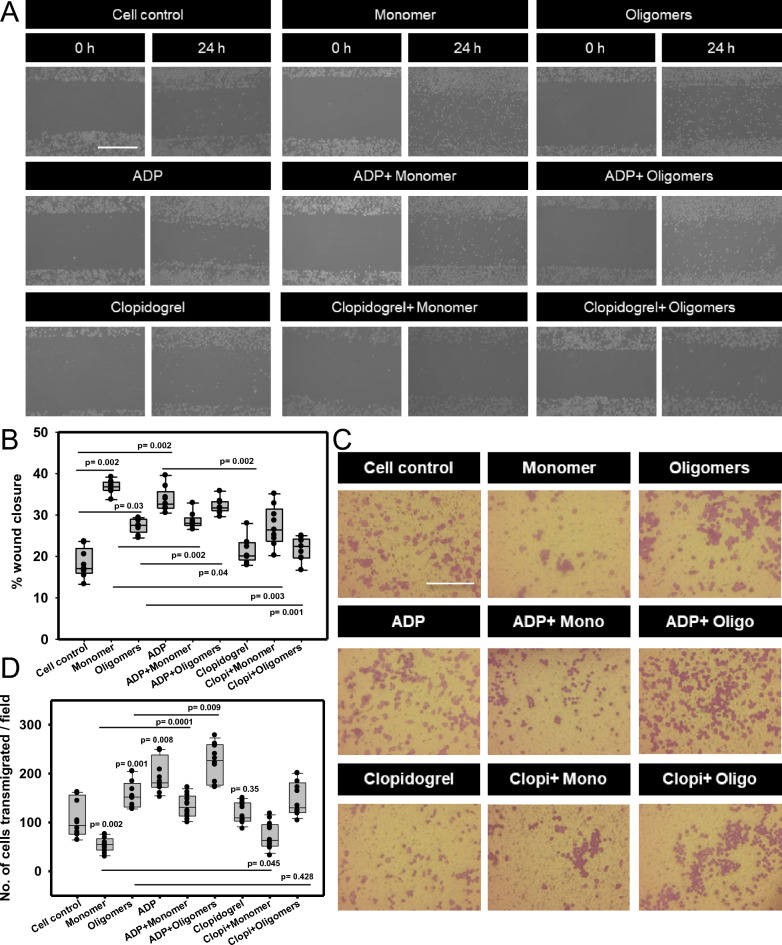
Extracellular Tau facilitates P2Y12 localization in filopodia and uropod, which degrades Tau deposits. **A** P2Y12 was colocalized with F-actin in filopodia and branched uropod of migratory microglia. Extracellular Tau oligomers exposure induced the localization of P2Y12 more in microglial filopodia and uropod as compared to monomer-treated cells. (Scale bar 10 μm) **B** The P2Y12^+^ filopodia^+^ microglia has significantly increased up to 80% upon Tau oligomers exposure than monomer exposure at 50%. (No. of experiment = 3) (n = 22). **C** P2Y12 intensity has increased in uropod by Tau oligomers and ADP exposure in migratory microglia (No. of experiment = 3) (n = 50). **D** The coverslips were coated with Tau monomer and oligomers to mimic the scenario of Tau depositions. The N9 microglia were seeded onto the coated coverslip to determine the potency of Tau plaques degradation. Microglia degraded Tau monomeric deposits by 8 h and Tau oligomers deposits by 24 h after seeding, through the formation of the F-actin-rich P2Y12^+^ podosome. It was evident that the degradation of Tau monomers was faster than oligomeric deposits (scale bar 10 μm). **E** The percentage of microglia, which degraded Tau monomer- deposits, was 38%, while 30% of microglia degraded Tau oligomers deposits. (No. of experiment = 3) (n = 40). **F** The P2Y12-associated podosome in microglia degraded Tau monomer deposits more than Tau oligomers-deposits. (No. of experiment = 3) (n = 30). **G** Microglia was also found to degrade Tau deposits through filopodia formation, which was orchestrated with P2Y12 (scale bar 10 μm). It was evident for the first time that P2Y12^+^ filopodia could degrade Tau monomer and oligomers deposits, which connects the signaling of chemotaxis and matrix degradation. **H** Microglia degraded the Tau deposits more in the case of the Tau monomer than the Tau oligomers deposits, through the formation of P2Y12^+^ filopodia. (No. of experiment = 3) (n = 30). **I** Extracellular Tau oligomers have induced the P2Y12-driven chemotaxis, which leads to the substratum adhesion and Tau deposit degradation through the formation of podosome and filopodia in migratory microglia

Our previous study showed that extracellular Tau oligomers act as a chemoattractants by interacting with microglial P2Y12 with elevated wound closure and trans-migration [22]. To identify the importance of P2Y12 signaling in Tau oligomers-induced migration, P2Y12 was activated and blocked by ADP and Clopidogrel, respectively, then the migration was measured in terms of %wound closure in microglial culture. We observed that both monomer and oligomers have induced the microglial migration by two times upon ADP-mediated P2Y12 activation as well as individual treatment conditions, as compared to untreated groups. While, the blockage of P2Y12 signaling has significantly reduced the %wound closure as compared to the ADP-treated group (Fig. 4A, B). However, the microglial migration has also been reduced upon the exposure of both the Tau species, when the cells were co-treated with Clopidogrel as compared with ADP (Additional file 1: Figure S4A). Next, we studied the trans-well migration of microglia in response to Tau monomer and oligomers along with ADP and Clopidogrel exposure. In comparison, we observed that the Tau oligomers exposure has resulted in increased microglial trans-migration, which was also evident in ADP + Tau oligomers-induced group (Fig. 4C). The P2Y12 blockage led to the reduced level of microglial trans-migration, but the Tau oligomers could reverse the rate of trans-migration even upon P2Y12-blocked conditions (Fig. 4D). Hence, it is evident that P2Y12 signaling can influence the microglial migration while the Tau oligomers have the potential to intervene in P2Y12-mediated chemotaxis to invade during disease conditions.

### Microglia orchestrate P2Y12^+^ podosome and filopodia to degrade more Tau monomer deposits than oligomers

**Fig. 5:**
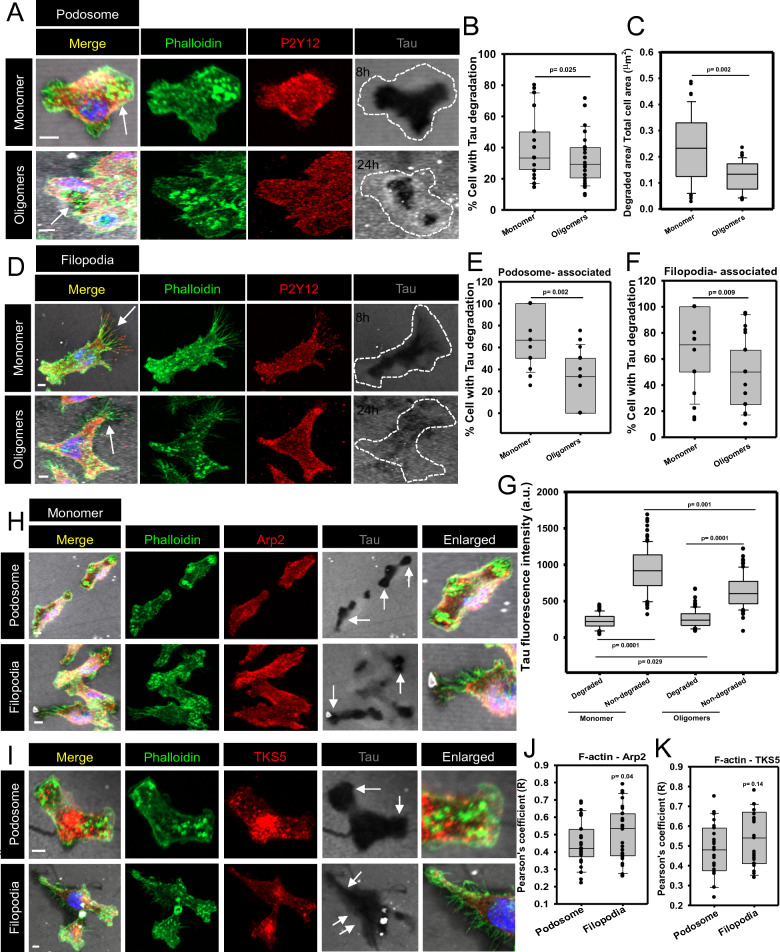
Microglia degrades Tau deposits by actin remodeling, localized with TKS5 and Arp2. **A** Microglia degraded Tau deposits through the accumulation of podosome and filopodia, which were localized with Arp2 actin nucleator at the site of degradation (Scale bar 10 μm). The arrow indicates the degradation area. **B** The actin remodeling is mediated by Arp2, where filopodia contained more Arp2 than podosome, relating to rapid actin polymerization at the Tau deposits degradation site (No. of experiment = 3) (n = 30). **C** Similarly, the TKS5 adaptor protein became colocalized with podosome and filopodia at the site of Tau deposits degradation (scale bar 10 μm). The arrow indicates the degradation area. D. But, the colocalization of F-actin and TKS5 did not alter between podosome and filopodia-associated Tau deposits degradation. (No. of experiment = 3) (n = 30) **E** Tau fluorescence intensity was significantly reduced in the degradation area of Tau monomer and oligomers-deposits as compared to the non-degraded area. Moreover, Tau monomer was significantly degraded more than oligomers in microglia-mediated deposit degradation (No. of experiment = 3) (n = 100). **F** The quantification of relative degraded area/total cell area revealed that monomer deposits were better degraded by microglia as compared to Tau oligomers deposits. (No. of experiment = 3) (n = 45). Hence, It can be concluded that microglia prefer to degrade Tau monomer more than oligomers as deposits as emphasized by Time and area of degradation

The activation of the integrin receptors and growth factor receptors (PDGF, EGF) results in the induction of the podosome formation by Src kinase and PKC in various migratory cells [50, 51]. But, the occurrence of the chemotactic receptor-like-purinergic P2Y12 in podosome is not yet described. Here, we observed the presence of P2Y12 in microglial podosome, which were associated with the degradation of Tau monomer and oligomers deposits (Fig. 5A). Moreover, we found that monomer deposits were degraded at 8 h of cell seeding while; the oligomers were not degraded at the same time point. For oligomers deposits, microglia started degradation after 24 h of cell seeding, but the monomer deposits were completely degraded at the 24 h time-point (Additional file 1: Figure S5A). Quantitatively, microglia preferred to degrade Tau monomers more than Tau oligomers as deposits where 41% and 33% of cells degraded Tau monomer and oligomers, respectively (Fig. 5B). The quantification of the relative Tau-degraded area (degradation area/Total cell area) depicted that the monomer deposits were significantly degraded more than the oligomers-deposits area (Fig. 5C). Filopodia are the important bundled actin structures that function in adhesion, mechano-sensing, directional migration and phagocytosis [52]. However, the functions of filopodia in the degradation of plaque deposits were not yet studied. In our study, we found for the first time that microglial filopodia decorated with P2Y12 were found to degrade the Tau deposit similar to podosome (Fig. 5D). Quantitatively, 72% of the microglia were associated with Tau monomer-deposit degradation, while only 26% of microglia could degrade Tau oligomers by forming podosome (Fig. 5E). Moreover, the filopodia-associated Tau-deposit degradation was mediated by 70% of microglia in the case of monomer, and 50% cells for oligomers deposit degradation (Fig. 5F). For further clarification, we have also quantified the Tau fluorescence intensity from degraded and non-degraded spots in the microglia. Here, we found that the Tau monomer and oligomers intensity were significantly reduced in Tau-degraded area as compared to non-degraded spots. Moreover, the monomer fluorescence was comparatively reduced from the oligomers intensity in the Tau-deposits degradation area (Fig. 5G). Hence, these results signify that P2Y12 signaling actively takes part in the regulation of podosome and filopodia-associated matrix adhesion as well as microglial migration to degrade Tau deposits.

### Microglia degrade Tau deposit by Arp2-decorated podosome and filopodia

The previous report has shown that P2Y12 activation is associated with alternatively activated microglia (M2), and the P2Y12^+^ microglia were located adjacent to the multiple sclerosis plaques [53]. In our study, microglial podosome and filopodia, which were associated with Arp2, were evidenced in Tau-deposit degradation (Fig. 5H, Additional file 1: Figure S5B). Similarly, microglial podosome and filopodia were observed to degrade the Tau deposit, colocalized with TKS5, responsible for podosome formation and matrix degradation (Fig. 5I, Additional file 1: Figure S5C). Colocalization analysis depicted that the filopodia were more orchestrated with Arp2 and F-actin than podosome during microglia-mediated deposit degradation (Fig. 5J). But, the TKS5 association with F-actin in podosome and filopodia remain unaltered during Tau degradation (Fig. 5K). Thus, it is evident that filopodia-mediated Tau degradation requires faster actin nucleation/polymerization than podosome-mediated degradation by migratory microglia. Hence, this signifies that microglia degraded Tau monomer deposits more efficiently than the oligomers deposits which may emphasize the complicated degradation/elimination of Tau oligomers and their concomitant toxicity in AD brain scenario.

### The blockage of P2Y12 signaling reduces Tau-deposit degradation

**Fig. 6:**
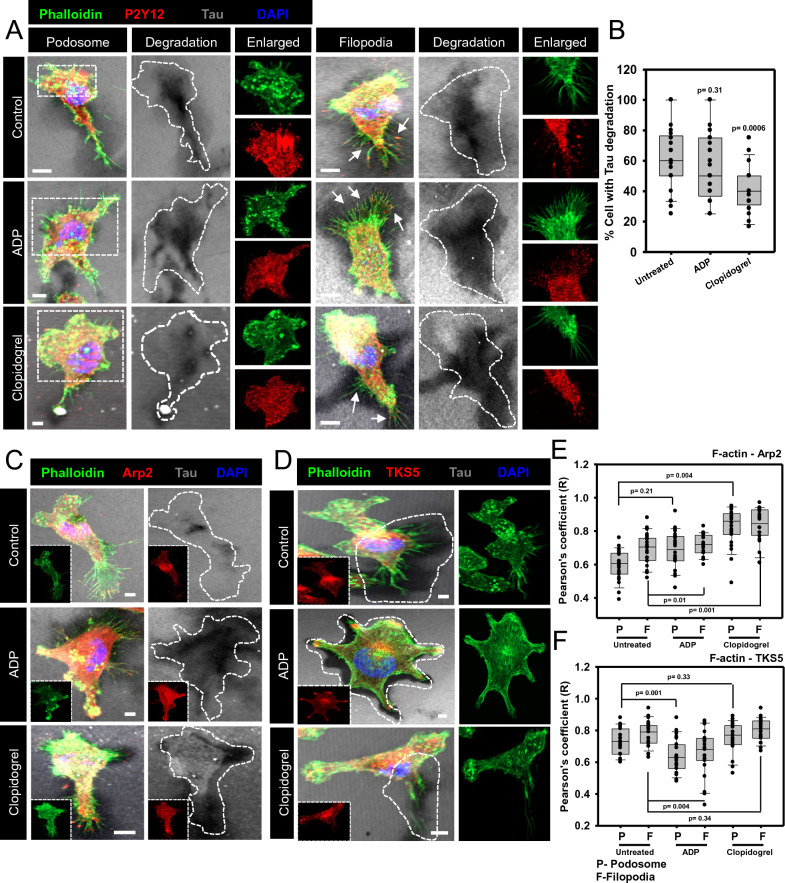
P2Y12-activation and blockage influence Tau deposit degradation through the Arp2-mediated podosome and filopodia formation. **A** P2Y12 activation by ADP has induced more filopodia formation for Tau deposit degradation. While, microglia, which degrade Tau, deposits by P2Y12.^+^ podosome formation, were independent of P2Y12 signaling activation/blockage. Microglia have been shown to degrade Tau deposits as scattered spots at the cell surface upon Clopidogrel treatment. While upon ADP exposure, microglia degraded the Tau deposits throughout the cell surface (Scale bar 10 μm). **B** The percentage of cells with Tau deposit degradation has been reduced by Clopidogrel exposure. While the ADP-mediated P2Y12 activation showed unaltered Tau deposit degradation compared to the control. (No. of experiment = 3) (n = 33). **C** P2Y12 activation resulted in more Arp2-localized filopodia formation that leads to Tau degradation, while Clopidogrel treatment did not alter the membrane-associated actin and Tau deposit degradation (Scale bar 10 μm). **D** Similarly, TKS5 localization was reduced in podosome and filopodia during Tau deposit degradation upon P2Y12-activated microglia (Scale bar 10 μm). **E** P2Y12 activation leads to the Arp2-associated filopodia formation at the site of Tau deposit degradation. At the same time, the blockage of P2Y12 signaling by clopidogrel resulted in more accumulation of Arp2-orchestrated podosome and filopodia at the Tau degradation site. (No. of experiment = 3) (n = 25). **F** ADP-mediated P2Y12 activation lead to the reduced TKS5 colocalization at podosome and filopodia while P2Y12 blockage did not alter TKS5 localization in remodeled actin network. (No. of experiment = 3) (n = 25)

Microglial P2Y12 dictates the directional migration via actin remodeling, and filopodia formation for matrix degradation by secreting various proteases to reach the site of neuronal damage [48, 54, 55]. In our study, the function of P2Y12 signaling was explored in Tau clearance by coating the coverslips with Tau, which mimicked the situation of the Tau-deposited area in the brain. The ADP-induced microglia showed increased filopodia formation during Tau degradation, but the blockage of P2Y12 signaling did not alter the level of podosome formation (Fig. 6A, Additional file 1: Figure S6A, B). Microscopic quantification elucidated that the blockage of P2Y12 signaling by clopidogrel has reduced the level of Tau-deposits degradation while ADP induction did not alter extracellular Tau degradation (Fig. 6B). Similarly, the podosome and filopodia were colocalized with Arp2 and TKS5 in Tau degradation area, which signifies actin nucleation and podosome turnover in microglia (Fig. 6C, D, Additional file 1: Figure S6C, D). The colocalization analysis depicted that P2Y12 blockage lead to more accumulation of Arp2 with F-actin. At the same time, the ADP-induced activation resulted in less TKS5 localization in podosome and filopodia in Tau-degrading microglia (Fig. 6E, F). Therefore, the influential function of microglial purinergic receptor P2Y12 can be decoded in directional chemotaxis and in the degradation of extracellular Tau deposits for therapeutic intervention.

## Discussion

Podosome are the short-lived actin protrusions at the ventral surface of the cells for mediating matrix anchorage, ECM degradation, and migration [54]. Podosome are remarkably different from other protrusive structures like lamellipodia and filopodia, based on the actin organization, cellular location, function and specific molecular signature [25, 51]. The podosome core contains F-actin with actin nucleator Arp 2/3 complex and TKS5 at podosome mediates the ECM degradation [56, 57]. The podosome, lamellipodia, and filopodia in immune cells function in the surveillance, tissue remodeling, ECM degradation, cytokine release, and antigen recognition [58–60]. Here, we emphasized that microglia sense the extracellular Tau oligomers and induce rapid actin nucleation by incorporating Arp2 in migration-associated structures such as podosome and filopodia. Moreover, the microglia skewed the turnover of actin-associated proteins- Arp2 and TKS5 in the podosome and filopodia at lamellipodia from the uropod for directional migration in Tau-induced conditions. Migratory cells form various rearrangements of podosome having unknown functions in the physiological state [51], *e.g.*, osteoclasts formed single podosome-rich arrangements termed ‘sealing zone’, which get matured into podosome belts for bone remodeling [61]. The podosome clusters are constantly formed by the fusion and fission of parent podosome for mechano-transduction for macrophage migration [62]. Similarly, podosome rosettes are formed in various cells by PKC, Rho-GTPase, and integrin signaling, mediating through phosphoinositide-(3,4)-P2 (PIP2) and N-WASP. The PIP2 and N-WASP, then, subsequently interact with TKS5 and Grb2 for migration and ECM degradation [12, 30, 63–65]. In our study, we investigated the appearance of different podosome arrangements in Tau-induced microglia. Extracellular Tau exposure was associated with the formation of various podosome structures such as podosome belts, clusters, and well-connected single podosome. Among all, the TKS5-localized clustered podosome were induced with the F-actin in Tau-exposed microglia. Hence, it is emphasized that extracellular Tau influences TKS5-associated podosome formation and clustering for matrix adhesion and degradation in migratory microglia.

Cell migration and invasion play a vital role in embryonic development, combating infection, and repairing injury. But the abnormal cellular migration resulted in carcinogenesis, immune disease, genetic disorders (Wiskott Aldrich Syndrome, Frank-Ter Harr Syndrome) and neurodegenerative disease-AD [51] [66]. The metabotropic P2Y12 signaling is mainly associated with homeostatic microglia, which involves in filopodia formation, maintaining neuronal health and chemotaxis. The activation of P2Y12 signaling collapses filopodia and induces large process extensions with bulbous tips, signifying the homeostatic and immune-surveilling microglia [48]. Moreover, the DAMs showed a reduced expression of various homeostasis genes, such as- P2Y12 and CX3CR1 in AD and Tauopathy mice models [67]. The P2Y12^+^ microglia were observed to surround the Aβ and Tau plaques in the Tauopathy mice brain [13]. But, the direct function of P2Y12 in the actin network of migratory microglia needs to be further explored [68, 69]. We hypothesized that the chemotactic microglia form lamellipodia and filopodia for migration and podosome for ECM degradation to mediate Ca^2+^ signaling, phagocytosis, and inflammation [14]. Later, we showed that microglial P2Y12 directly interacts with extracellular Tau oligomers and induces the remodeling of membrane-associated actin structures [22, 41]. Here, we particularly elucidated that the P2Y12 receptor was localized with F-actin-rich podosome and filopodia in response to extracellular Tau oligomers. This co-occurrence may emphasize the coupling of microglial chemotaxis and podosome formation to chase and eliminate extracellular Tau oligomers from the microenvironment. It has been previously shown that the P2Y12 interacts with β1-integrin to mediate chemotaxis and tissue invasion, while the blockage of P2Y12 leads to reduced microglial migration, pro-inflammatory cytokine production and neuroprotection in ischemic stroke [70, 71]. In our study, we evidenced that both the Tau monomers and oligomers have induced the microglia-mediated wound closure, while, the activation and blockage of P2Y12 signaling directly influence the rate of migration in Tau-induced microglia. Similarly, Tau oligomers have increased the invasion in ADP-induced microglia. But, the trans-migration was reduced upon the blockage of P2Y12 signaling, and restored moderately by Tau oligomers treatment. Hence, it signifies that P2Y12 signaling has a direct influence on microglial chemotaxis and tissue invasion, while the extracellular Tau oligomers can significantly intervene in the P2Y12 signaling and migration in the Tauopathy condition.

**Fig. 7:**
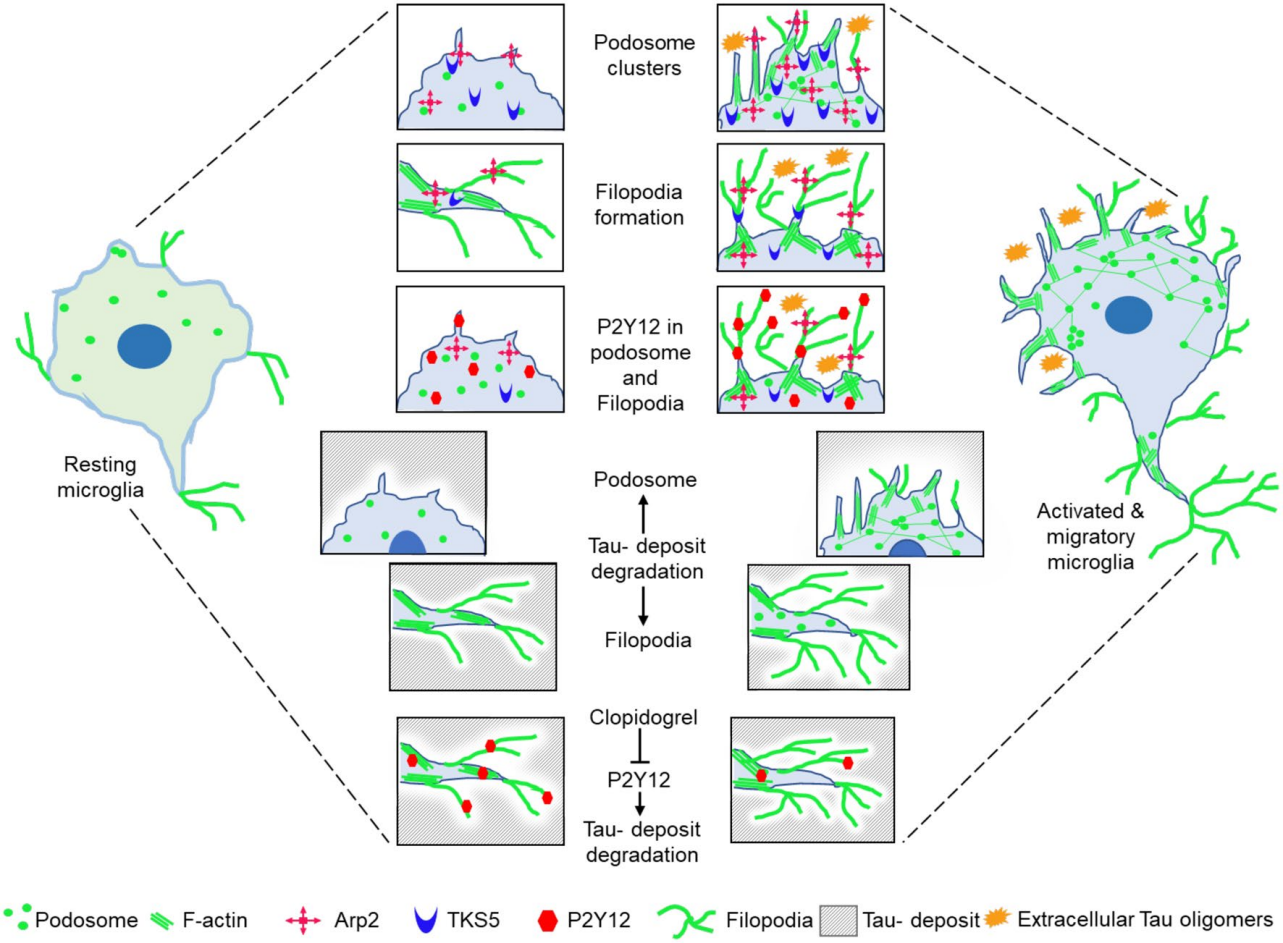
Microglia degrade extracellular Tau deposits by P2Y12-mediated podosome and filopodia formation. Extracellular Tau oligomers have induced Arp2 and TKS5-associated podosome clusters and filopodia formation during P2Y12-mediated microglial migration and invasion. Moreover, Tau monomer and oligomers deposits can be degraded by microglial filopodia and podosome formation. The blockage of P2Y12 signaling has reduced the microglial chemotaxis and P2Y12-associated filopodia formation and Tau-deposits degradation. Hence, P2Y12 signaling plays a dual role in extracellular Tau-induced microglial chemotaxis and the clearance of Tau deposits via podosome and filopodia-associated actin remodeling

Similar to cancer cells, smooth muscle cells, endothelial cells, and immune cells show podosome-mediated ECM degradation and cell migration [72–74]. Similarly, human macrophages, dendritic cells and lymphocytes form long podosome protrusions for matrix-degradation when cultured in fibrillar collagen gel [73, 75]. The genetic knockdown of TKS5 inhibits the podosome formation while the loss of TKS4 affects both podosome formation and MT1-MMP9-associated ECM degradation [57, 76]. In our study, we showed that microglia degrade the extracellular Tau deposits by Arp2- and TKS5-associated podosome and filopodia formation. The previous report has shown that phagocytic microglia mediate oxidative injury, antigen presentation, and T-cell activation in the active lesion of multiple sclerosis. While during the resolving stages, microglia transformed back into P2Y12^+^ TMEM119^+^ phenotype in the inactive plaque-associated region [77]. Similarly, we mimicked the plaque deposition scenario by immobilizing Tau onto the coverslip and then allowing the microglia for degrading the Tau deposit. We found that microglia eventually degrade Tau deposits through the formation of podosome and filopodia orchestrated with the P2Y12 receptor. Interestingly, microglia efficiently degrades Tau monomers more than oligomers deposits, as observed by the prolonged periods (8 and 24 h) for oligomers degradation with the lesser area and fluorescence intensity of degraded Tau. Moreover, the blockage of P2Y12 signaling resulted in the reduction of Tau degradation. Hence, it can be stated that microglial P2Y12 can mediate the directed migration and also involves Tau deposit degradation by remodeling the actin network. Extracellular Tau oligomers influence the Arp2 and TKS5-associated podosome and filopodia formation through the activation of P2Y12 signaling for microglial migration. The formation of podosome clusters and filopodia may dictate the degradation of Tau- deposits by involving P2Y12 signaling. Hence, the P2Y12 pathway contributes a dual function in extracellular Tau oligomers-induced chemotaxis and the clearance of Tau- deposits via podosome and filopodia-associated actin remodeling (Fig. 7).

## Supplementary Information

**Additional file 1: Figure S1.** Preparation and characterization of Tau oligomers, Tau oligomers induced Arp2- associated actin remodeling in microglia. **Figure S2.** Extracellular Tau induced the accumulation of various podosome rearrangements as single, belt and clusters in migratory microglia. **Figure S3.** Extracellular Tau oligomers facilitate podosome clusters in time-dependent manner, localized with P2Y12. **Figure S4.** Tau oligomers induced microglial migration, mediated of P2Y12 signaling. Figure S5. Microglia degraded Tau deposits by P2Y12, Arp2 and TKS5-accumulated podosome and filopodia. **Figure S6.** Microglia degraded Tau deposits which is reduced by P2Y12 antagonist, clopidogrel, localized with Arp2 and TKS5.

## Data Availability

All data supporting the conclusions of this article are included within the article and in additional information files are provided.

## Abbreviations

AD: Alzheimer’s disease
MTs: Microtubules
MTOC: Microtubule organizing center
P2Y12: Purinergic receptor 12
MAPs: Microtubule-associated proteins
TIPs: Microtubule plus end-binding proteins
