# Supplementary material for: Microglia degrade Tau oligomers deposit via purinergic P2Y12-associated podosome and filopodia formation and induce chemotaxis
Source: Cell Biosci. 2023 May 23;13:95. doi: 10.1186/s13578-023-01028-0 (PMC10204346; doi:10.1186/s13578-023-01028-0)
Supplement: Supplementary file 1 — Additional file 1: Figure S1. Preparation and characterization of Tau oligomers, Tau oligomers induced Arp2- associated actin remodeling in microglia. Figure S2. Extracellular Tau induced the accumulation of various podosome rearrangements as single, belt and clusters in migratory microglia. Figure S3. Extracellular Tau oligomers facilitate podosome clusters in time-dependent manner, localized with P2Y12. Figure S4. Tau oligomers induced microglial migration, mediated of P2Y12 signaling. Figure S5. Microglia degraded Tau deposits by P2Y12, Arp2 and TKS5-accumulated podosome and filopodia. Figure S6. Microglia degraded Tau deposits which is reduced by P2Y12 antagonist, clopidogrel, localized with Arp2 and TKS5. [file 13578_2023_1028_MOESM1_ESM.pdf]

Supplementary Information

Microglia degrade Tau oligomers deposit via purinergic P2Y12-associated podosome and filopodia formation and induce chemotaxis

Subashchandrabose Chinnathambi [1, 2, *] and Rashmi Das [1, 2]

[1]Neurobiology Group, Division of Biochemical Sciences, CSIR-National Chemical Laboratory, Dr. Homi Bhabha Road, Pune- 411008, India

[2]Academy of Scientific and Innovative Research (AcSIR), Ghaziabad, 201002, India

3Department of Neurochemistry, National Institute of Mental Health and Neuro Sciences (NIMHANS), Institute of National Importance, Hosur Road, Bangalore -560029, Karnataka, India.

[*]To whom correspondence should be addressed**: Prof. Subashchandrabose Chinnathambi**, Neurobiology group, Division of Biochemical Sciences, CSIR-National Chemical Laboratory (CSIR-NCL), Dr. Homi Bhabha Road, 411008 Pune, India, Telephone: +91-20-25902232, Fax. +91-20-25902648. Email: **s.chinnathambi@ncl.res.in.** Department of Neurochemistry, National Institute of Mental Health and Neuro Sciences (NIMHANS), Hosur Road, Bangalore -560029, Karnataka, India. Email: **subashneuro@nimhans.ac.in**

Authors email address

Rashmi Das: rd.das@ncl.res.in

Subashchandrabose Chinnathambi: s.chinnathambi@ncl.res.in; subashneuro@nimhans.ac.in

Supplementary Figure 1

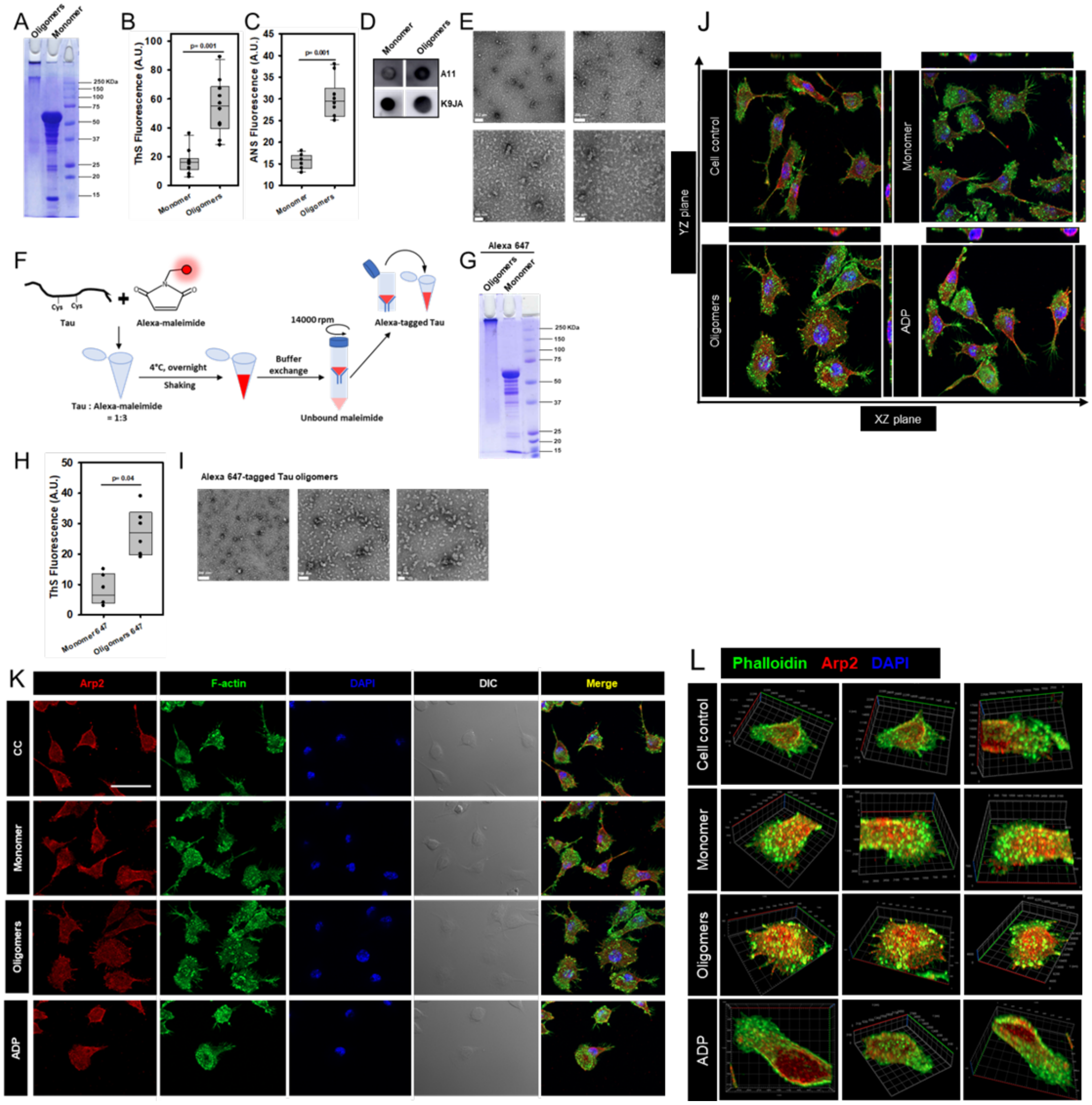

Supplementary Fig 1: Preparation and characterization of Tau oligomers, Tau oligomers induced Arp2-associated actin remodeling in microglia. A. Tau oligomers corresponded to higher molecular weight than Tau monomer in SDS-PAGE. B,C. Tau monomers showed less ThS and ANS fluorescence than Tau oligomers. D. Tau oligomers were characterized by Dot blot as A11 antibody recognized oligomers more than monomer in comparison with total Tau K9JA antibody. E. Transmission electron microscopy revealed the heterogenous globular Tau oligomers. F. Tau and alexa^{647}-maleimide were mixed with 1:3 ratios and incubated overnight for Alexa^{647}-tagged Tau. G. Alexa^{647}-tagged Tau monomer and oligomers were characterized by SDS-PAGE. H, I. Alexa^{647}-tagged Tau oligomers have more ThS fluorescence and globular structures as observed by TEM. J, K. Tau oligomers induced the Arp2-induced membrane-associated actin polymerization, similar to ADP exposure. Orthogonal projection through XZ and YZ plane confirmed the presence of Arp2-localized F-actin at the cortical layer of cell in Tau- and ADP-induced microglial migration. Scale bar: 100 µm. L. The 3D microscopic images in various rotation angles revealed that the accumulation of Arp2-associated podosome in microglial lamella by extracellular Tau oligomers exposure than Tau monomer.

Supplementary Figure 2

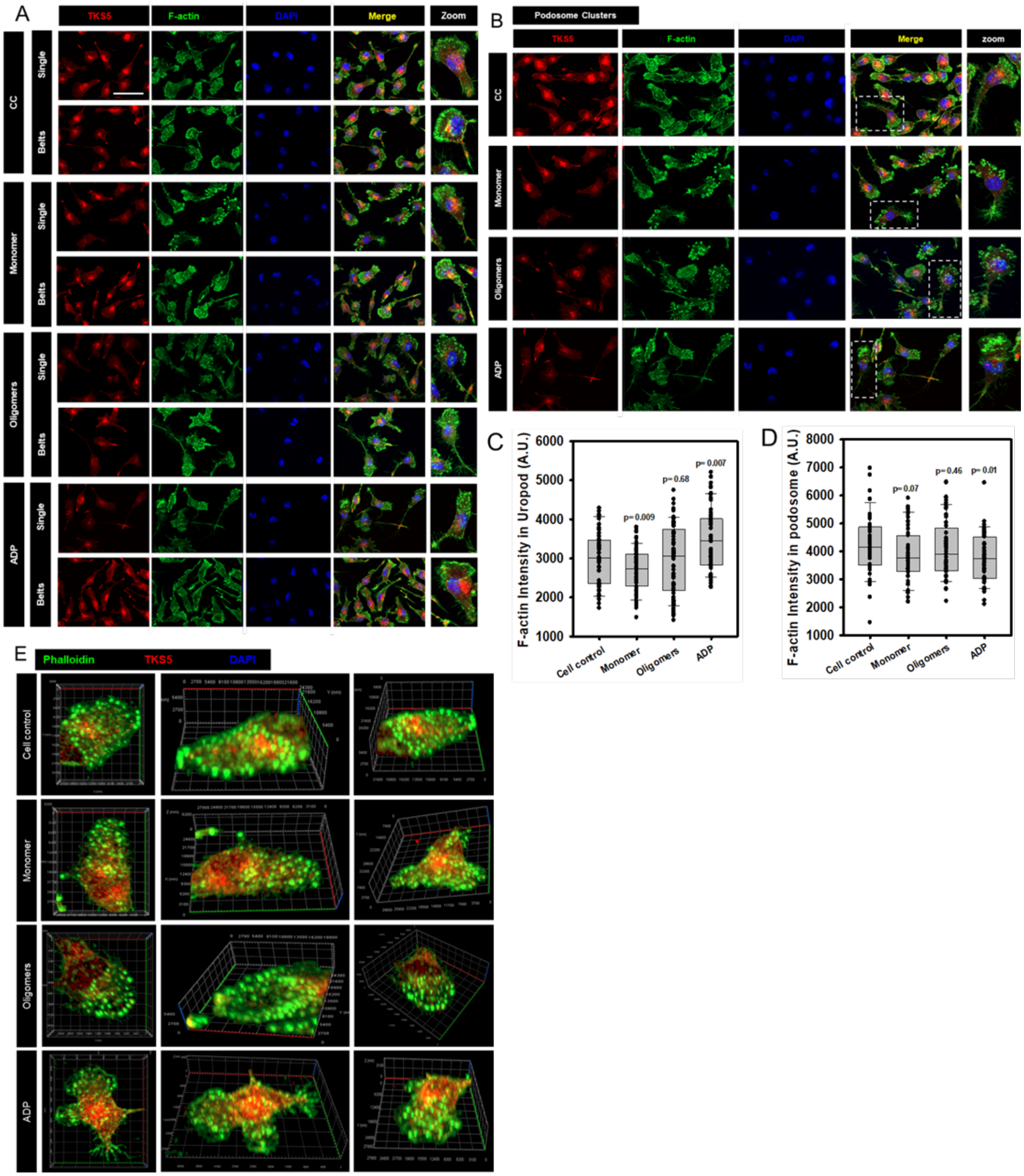

Supplementary Fig. 2. Extracellular Tau induced the accumulation of various podosome rearrangements as single, belt and clusters in migratory microglia. A. Tau monomer and oligomers induced the accumulation of TKS5-localized single and podosome belts in the lamellipodia of migratory microglia, Scale bar: 100 µm. B. Tau oligomers have induced the accumulation of podosome clusters in the growing lamellipodia for active microglial migration similar to ADP-mediated P2Y12 activation. Scale bar: 100 µm. C. The F-actin intensity in microglial uropod decreased in Tau monomer exposure and increased in the ADP-treated group in migratory microglia. D. F-actin intensity in podosome remains unaltered by Tau exposure but decreased in ADP-induced

migratory cells. E. The 3D microscopic images revealed that actin-ring-like podosome arrangement in the cellular cortex in Tau oligomers exposed cells as compared to Tau monomer.

Supplementary Figure 3

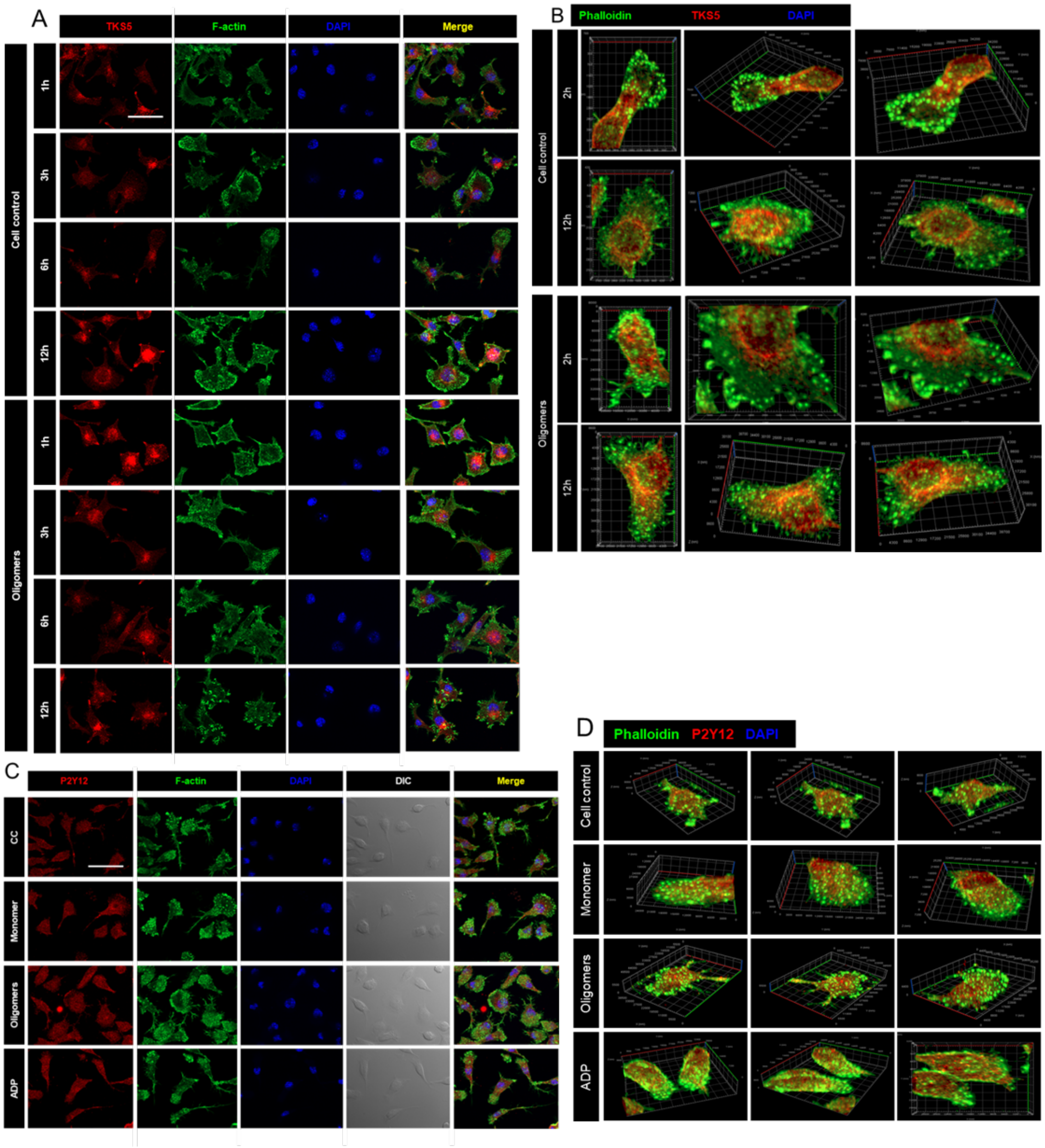

Supplementary Fig. 3. Extracellular Tau oligomers facilitate podosome clusters in time-dependent manner, localized with P2Y12. A. Tau oligomers exposures have induced the TKS5-driven podosome clusters accumulation (at 1hour) for active migration in a time-dependent manner as compared to cell control. Scale bar: 100 µm. B. The 3D microscopic images revealed the accumulation and distribution of podosome in microglial lamella in a time-dependent manner, upon Tau oligomers-exposure. C. Extracellular Tau monomer and oligomers have induced the P2Y12-associated podosome and filopodia accumulation in migratory microglia. Scale bar: 100 µm. D. The 3D microscopic images have shown that the formation of a single podosome in microglial lamella upon extracellular Tau monomer exposure, while, Tau oligomers have induced localization of P2Y12 in clustered podosome.

Supplementary Figure 4

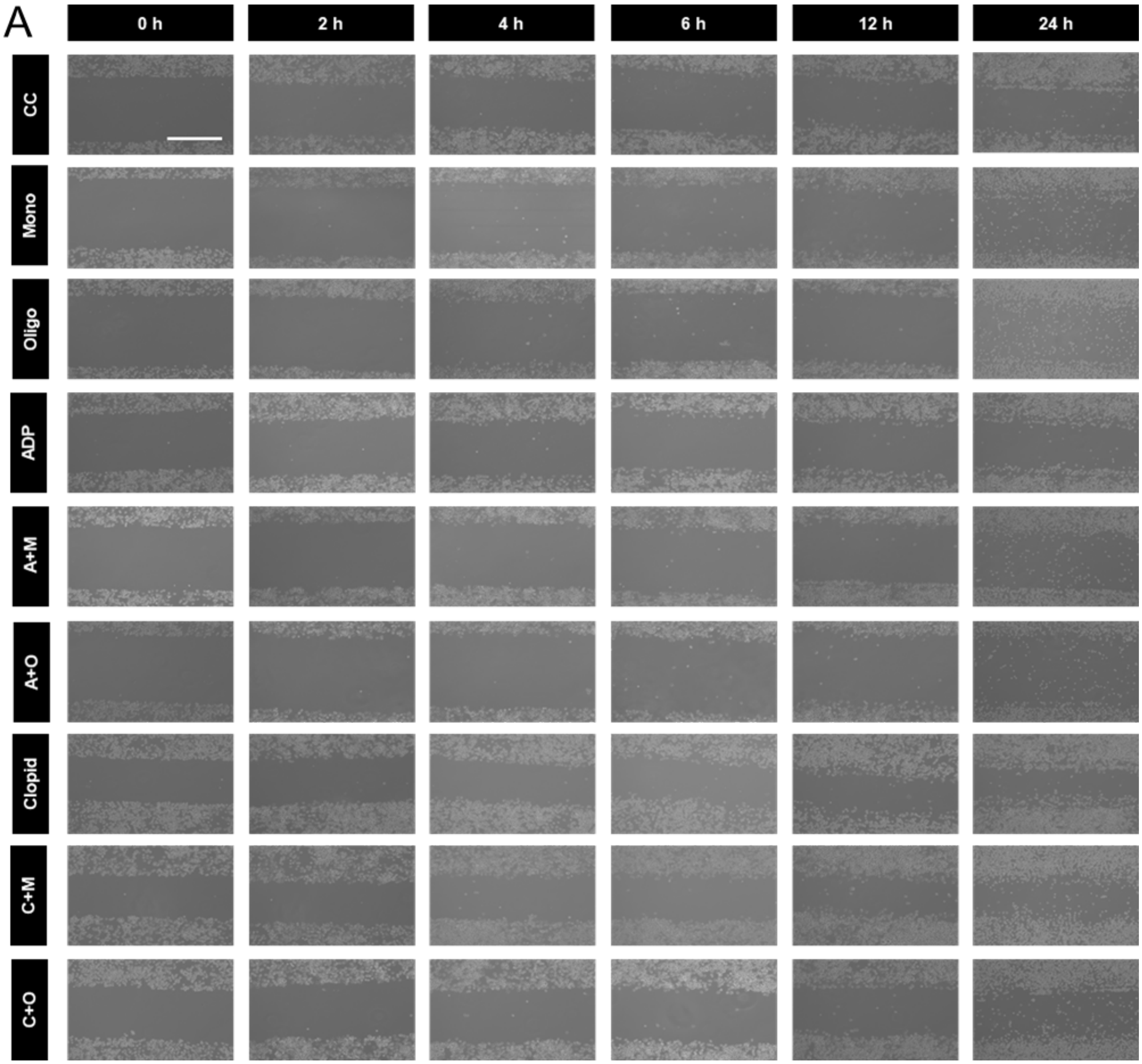

Supplementary Fig. 4. Tau oligomers induced microglial migration, mediated of P2Y12 signaling. A. Tau oligomers have induced microglial migration as compared to P2Y12 activation by ADP. While the P2Y12-blockage by Clopidogrel has reduced microglial migration as observed by 2D wound closure assay. Scale bar: 100 µm.

Supplementary Figure 5

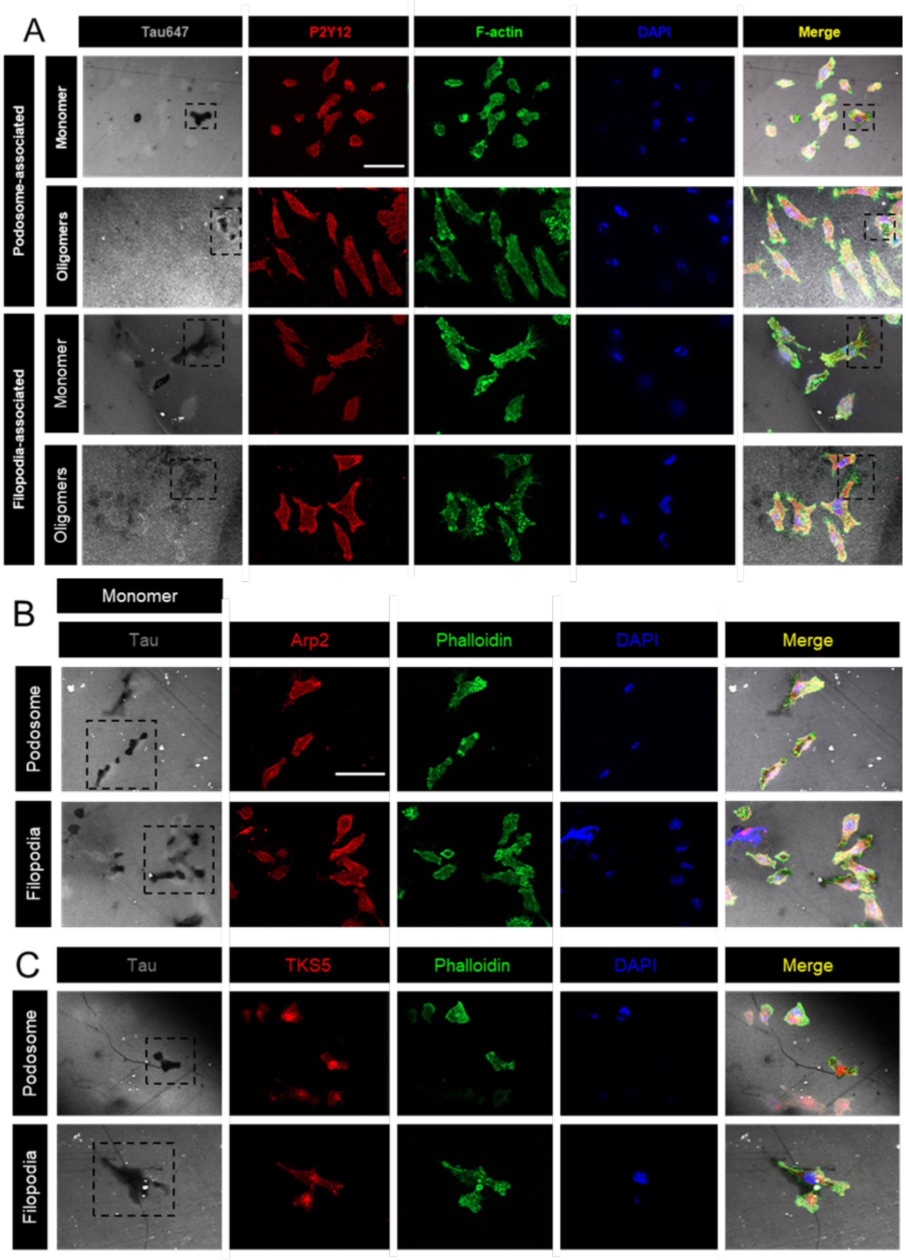

Supplementary Fig. 5. Microglia degraded Tau deposits by P2Y12, Arp2 and TKS5-accumulated podosome and filopodia. A. Tau monomers were found to be degraded more by N9 microglia as compared to Tau oligomers. P2Y12 become localized in podosome and filopodia, which were found to be associated with Tau, deposits degradation. The hollow spots were corresponding to the Tau degradation, which were localized with microglial podosome and filopodia. B. Microglia degrades Tau monomer as deposit by the accumulation of podosome and filopodia, among which filopodia were colocalized more with Arp2. C. The TKS5-associated F-actin-rich podosome were found to degrade Tau deposits more than filopodia in microglia. Scale bar: 100 µm.

Supplementary Figure 6

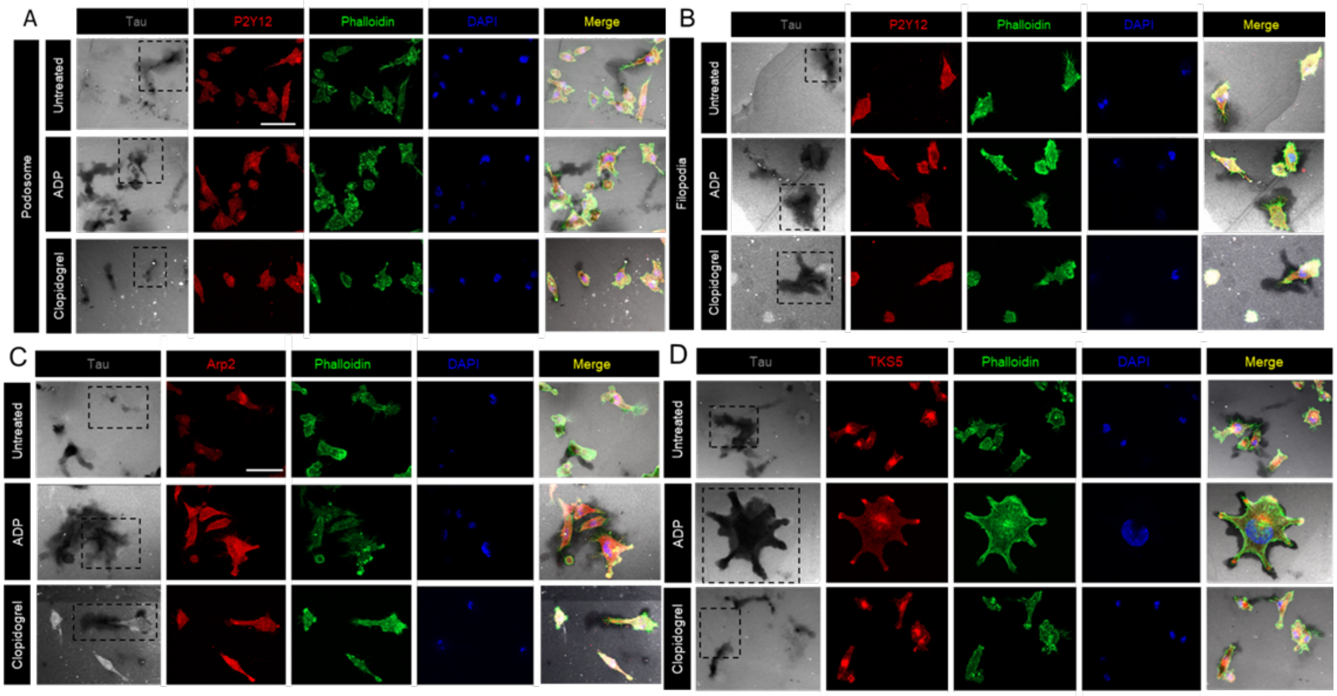

Supplementary Fig. 6. Microglia degraded Tau deposits which is reduced by P2Y12 antagonist, clopidogrel, localized with Arp2 and TKS5. A. ADP exposure did not alter the P2Y12-associated Tau degradation compared to control, while the clopidogrel exposure has reduced the Tau degradation by podosome formation. B. The P2Y12-localized filopodia-associated degradation of deposit was induced more in case of ADP exposure than a Clopidogrel-mediated purinergic blockage. C. ADP has induced microglial ECM degradation that are colocalized with Arp2 through P2Y12 activation. D. Similarly, TKS5 became more colocalized at the degradation area by P2Y12 signaling induction, while, P2Y12 blockage was associated with reduced Tau deposits degradation. Scale bar: 100 µm.
